# Gut–Liver Axis Derangement in Non-Alcoholic Fatty Liver Disease

**DOI:** 10.3390/children4080066

**Published:** 2017-08-02

**Authors:** Marco Poeta, Luca Pierri, Pietro Vajro

**Affiliations:** 1 Pediatrics, Department of Medicine, Surgery and Dentistry “Scuola Medica Salernitana”—University of Salerno, 84081 Baronissi (Salerno), Italy; m.poeta@outlook.it (M.P.); luca.pierri@hotmail.com (L.P.); 2 Pediatrics Residency Joint Programs, University of Naples Federico II, 80131 Naples, Italy & University of Salerno, 84081 Baronissi (Salerno), Italy

**Keywords:** children, endotoxin, gut–liver axis, GLA, intestinal permeability, microbiota, non-alcoholic fatty liver disease, NAFLD, non-alcoholic steato-hepatitis, NASH, pediatrics, probiotics

## Abstract

Non-alcoholic fatty liver disease (NAFLD) is the most frequent type of chronic liver disease in the pediatric age group, paralleling an obesity pandemic. A “multiple-hit” hypothesis has been invoked to explain its pathogenesis. The “first hit” is liver lipid accumulation in obese children with insulin resistance. In the absence of significant lifestyle modifications leading to weight loss and increased physical activity, other factors may act as “second hits” implicated in liver damage progression leading to more severe forms of inflammation and hepatic fibrosis. In this regard, the gut–liver axis (GLA) seems to play a central role. Principal players are the gut microbiota, its bacterial products, and the intestinal barrier. A derangement of GLA (namely, dysbiosis and altered intestinal permeability) may promote bacteria/bacterial product translocation into portal circulation, activation of inflammation via toll-like receptors signaling in hepatocytes, and progression from simple steatosis to non-alcoholic steato-hepatitis (NASH). Among other factors a relevant role has been attributed to the farnesoid X receptor, a nuclear transcriptional factor activated from bile acids chemically modified by gut microbiota (GM) enzymes. The individuation and elucidation of GLA derangement in NAFLD pathomechanisms is of interest at all ages and especially in pediatrics to identify new therapeutic approaches in patients recalcitrant to lifestyle changes. Specific targeting of gut microbiota via pre-/probiotic supplementation, feces transplantation, and farnesoid X receptor modulation appear promising.

## 1. Introduction

Non-alcoholic fatty liver disease (NAFLD), the hepatic manifestation of metabolic syndrome (MetS), is the most frequent form of chronic liver disease worldwide. Liver damage due to lipid accumulation in hepatocytes is a direct consequence of obesity (OB) and insulin-resistance (IR) pandemics related to adoption of a hypercaloric Western diet (WD) rich in saturated fats, meat and refined sugars and poor in vegetables, fruits and fish, along with sedentary lifestyle [1]. Nowadays it represents a major health concern in pediatric population also, where it can affect10% of children, and up to 70% of those who are obese [2]. Probably due to different sex-specific fat metabolism patterns [3], NAFLD distribution appears to be higher in obese male (35.3%) vs. female (21.8%) adolescents [4]. The histological spectrum of NAFLD includes simple hepatic steatosis and its chronical evolution patterns: non-alcoholic steatohepatitis (NASH) with inflammation and fibrosis ending into cirrhosis [5,6]. Although the development of more severe histological pictures up to end-stage liver disease has been described generally later in life, liver transplantation may be already needed in young adulthood [7].

A “multiple-hit” hypothesis may explain NAFLD pathogenesis and progression. In particular in the last few years, a growing interest has been devoted to gut–liver axis (GLA) dysfunction (i.e., intestinal dysbiosis, bacterial overgrowth, alteration of mucosa permeability) as a relevant second hit in NAFLD progression and therefore as possible alternative therapeutic target in those patients unable to get benefits deriving from lifestyle modification, healthy diet and physical activity promotion [1,7]. Objectives of our mini-review are to appraise: (1) the pathophysiology definition of GLA components; (2) the identification of GLA derangements involved in NAFLD pathogenesis and progression; and (3) the delineation of therapeutic perspectives via GLA modulation to prevent and/or treat obesity-related liver disease.

## 2. Methods

A literature search was performed in the PubMed database using the following MeSH (Medical Subject Headings) terms: “NAFLD” OR “Non-alcoholic fatty liver disease” OR “NASH” OR “Non-alcoholic steatohepatitis” AND “Gut-liver axis” OR “gut microbiota” OR “probiotics” OR “intestinal permeability”. Non-English literature was excluded. Most relevant papers derived from literature search were selected from authors to writing this mini-review.

## 3. Gut–Liver Axis

The strong anatomical and functional interaction between the gastrointestinal tract and liver defines the term GLA. This intimate connection is already expressed in embryogenesis by the common origin from the ventral foregut endoderm [8]. GLA is characterized by bidirectional traffic. Nutrients and factors derived from gut lumen reach the liver through portal circulation; bile acids produced by hepatocytes are released in the small intestine through the biliary tract. However, this description is simplistic because GLA does not only have a nutritional function. The axis is a complex structure and the alteration, in particular, of two of its components (intestinal barrier and gut microbiota) seems to play a key role in liver damage and progression [9].

The intestinal barrier is composed of: (1) the columnar epithelium of cells bound together by an apical junction complex (tight junctions (TJs) and zonula adherens), gap junctions, and desmosomes; (2) the mucus layer on the epithelial surface consisting of mucin molecules produced by goblet cells; and (3) the antimicrobial defenses provided by Paneth cells, and gut-associated lymphoid tissue (GALT) [10].

**Figure 1:**
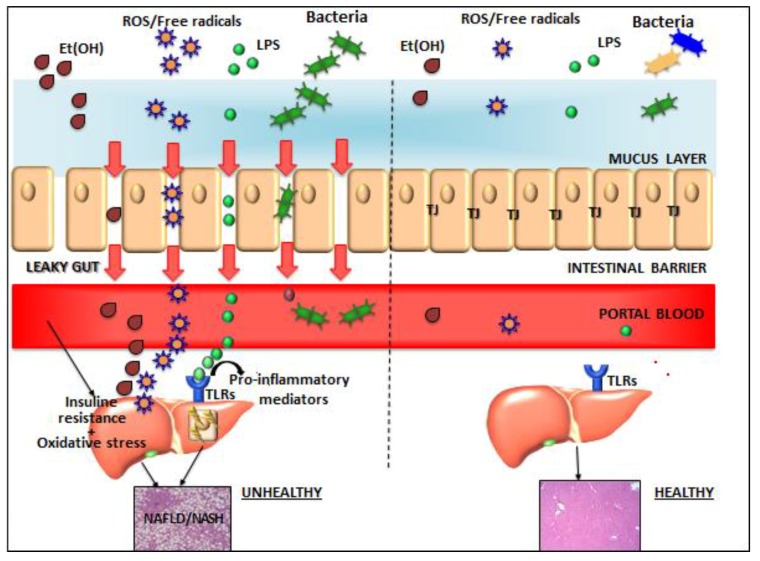
Gut–liver axis components in normal conditions (right part) and in non-alcoholic fatty liver disease (NAFLD)(left part).The presence of the dysbiotic microbiome and an altered intestinal barrier influenced by bacterial ethanol (EtOH), possibly associated with disruption of tight-junctions (TJs) (“leaky gut”), promotes the translocation of several bacterial products into the portal circulation. The interaction of bacterial products with toll-like receptors (TLRs) on the hepatic cell surface promotes the progression from simple steatosis to inflammation and fibrosis of the liver. ROS: reactive species of oxygen; NASH: non-alcoholic steatohepatitis; LPS: lipopolysaccharide; TLR: toll-like receptor.

The intestinal lumen is naturally colonized by the gut microbiota (GM), consisting of trillions of microorganisms from more than 1000 species, with a total weight of approximately 1–2 kg. As shown in Figure 1, GM has numerous health benefits, and in normal conditions, small amounts of bacterial products enter the liver via portal circulation and most of them are eliminated by hepatic macrophages called Kupffer cells (KCs). In presence of an intestinal dysbiosis and a break of intestinal barrier, it happens that increased bacteria/bacterial products translocation interrupts this immunological tolerance and promotes liver inflammation via toll-like receptor (TLR) stimulation [8].

However, also the liver may influence the microbiome according to the bidirectional definition of GLA. Bile acids in fact may have direct effects on bacteria causing membrane and/or DNA damage. Conversely, secondary molecules derived from bile acids metabolized by GM may activate specific host receptors (i.e., the farnesoid X receptor (FXR)) [11,12].

Bile contains immunoglobulin A (IgA) antibodies released by Peyer’s Patches of biliary submucosa capable of modulating the intestinal microbial load. Furthermore, IgA production is favorably influenced by normal microflora, with health benefits towards infections [13].

GM composition and GLA may therefore influence the antigenic traffic through intestinal barrier and the development of a series of diseases concerning a growing number of extra-intestinal organs [14], including hepatopathies, allergic diseases, diabetes mellitus type 1, familial Mediterranean fever, autism, cardiovascular disease, and dysfunctions in bone mass and immune response [15]. Here we will focus on the role of GLA components in NAFLD pathogenesis and progression.

## 4. Gut Microbiota

The GM is composed of bacteria, archea, virus, and fungi. Bacteria are dominated by four main phyla of bacteria (Firmicutes, Bacteroidetes, Actinobacteria, and Proteobacteria) which represent more than 95% of the total [10]. The GM is enriched in several genes important for glycan and aminoacid metabolism, xenobiotic metabolism, methanogenesis, and biosynthesis of vitamins [16]. This may explain why GM contributes to host nutrition, bone mineral density, modulation of the immune system, xenobiotics metabolism, intestinal cell development and proliferation, and protection against pathogens [17].

The GM is specific to an individual, and is also highly resilient, promptly returning to baseline after perturbation [18,19,20]. Interestingly, despite the unique individual GM, humans share similar functional gene profiles, implying a core functional microbiome [19].

Among several mechanisms which may explain the interaction between altered GM and obesity/NAFLD there are: (1) the augmented energy extraction from diet through the GM capacity to digest complex polysaccharides with consequent fermentation to short-chain fatty acids (SCFAs); (2) the intestinal epithelium damage mediated by bacterial ethanol production; (3) the bacterial/endotoxin translocation into the portal circulation and consequent liver damage via TLR signaling; (4) the modulation of bile acid (BA) synthesis; and (5) the reduction of choline metabolism with consequent decrement of very low density lipoproteins (VLDL) liver export [21].

The first clue on the role of the microbiota in the pathogenesis of obesity and NAFLD came from Backhed’s group studies [22]. They compared body weight gain in germ-free mice and conventionally raised mice, and found that the latter group gained more weight, with increased adipose tissue and body fat percentage, which could not be explained by different diet intake [22]. In two studies it has been shown that obese and overweight adults are more likely to have a low microbial gene count (LGC) than non-obese adults. LGC subjects were found to gain more weight over time and experience increased IR, dyslipidemia and elevated inflammatory markers. However, antibiotic treatments, type of diet and some food supplements should also be considered [23,24]. Taking into account several (and sometimes contradictory) studies in general, the link between intestinal dysbiosis and obesity/NAFLD is characterized by a greater prevalence of Firmicutes and a lower prevalence of Bacteroidetes in obese vs. lean individuals, and by a lower prevalence of Bacteroidetes in NASH patients vs. obese without NASH [21].

The microbiota can modulate body weight through several mechanisms. One is the fermentation pathway of indigestible carbohydrates in SCFAs: butyrate, propionate, and acetate [25]. Overall, colonic-derived SCFAs account for 10% of harvested energy from the diet, with acetate being the main source of energy [26]. Butyrate and propionate are considered anti-obesogenic, and acetate mainly obesogenic. Interestingly, while acetate and propionate are mainly produced by the phylum Bacteroidetes, butyrate is mainly produced by Firmicutes [27]. Butyrate is a major source of energy for colonocytes, increasing intestinal health and potentially modulating gut permeability and preventing metabolic endotoxemia [28]. Butyrate also seems to positively affect insulin sensitivity through stimulation of the release of the incretins glucagon-like peptide-1 (GLP-1) and gastric inhibitory polypeptide (GIP) [29]. Both butyrate and propionate can increase the expression of the anorexigenic adipokine leptin [30]. On the other hand, acetate is the most absorbed SCFA, and is a substrate for lipogenesis and cholesterol synthesis in the liver and adipose tissue [28]. Finally, SCFAs bind to specific receptors in the gut, liver, and adipose tissue, where they seem to have anti-inflammatory and metabolic actions that protect from obesity [26]. Interestingly, supplementation of oral butyrate in mice fed a WD, partially prevented liver steatosis and inflammation, while having no effect on obesity [31].

GM can also decrease the intestinal expression of the adipose tissue lipoprotein lipase inhibitor fasting induced adipose factor (FIAF). The result is an increased uptake of fatty acids in the adipose tissue and liver, favoring expansion of the adipose tissue and hepatic steatosis. Microbiota also prevent the beneficial action of FIAF in the expression of peroxisome proliferator-activated receptor (PPAR)-1α coactivator (PGC) and fatty acid oxidation [32]. Other mechanisms by which GM promotes obesity are an increase in mucosal gut blood flow enhancing nutrient absorption [33]; inhibition of adenosine monophosphate-activated protein kinase (AMPK) in the liver and muscle, leading to peripheral fatty acids oxidation and insulin resistance; and modulation of the pattern of conjugated bile acids including their function in lipid absorption [34].

A recent meta-analytic study shows a possible association between *Helicobacter pylori* (*H. pylori*) infection and NAFLD in adults [35]. *H. pylori* seems to promote metabolic variations, which are considered NAFLD risk factors, including IR, systemic inflammation and dyslipidemia [36]. Furthermore, presence of *H. pylori* may induce gastric atrophy, with consequent acid losses predisposing small intestinal bacterial overgrowth (SIBO), leaky gut and portal endotoxin translocation. A recent cohort study of 17,028 adults without NAFLD at baseline showed a significant association of *H. pylori* infection and the development of NAFLD, independent of metabolic and inflammatory risk factors [37], but another study including 3663 adults showed inverse results without a significant correlation [38]. Because of these contrasting results and of therapeutic potential extensive longitudinal studies at all ages including childhood are therefore needed to confirm the pathogenetic role of *H. pylori*.

## 5. Intestinal Permeability

The liver has both an arterial and a venous blood supply, with the greatest part of hepatic blood flow coming from the gut via the portal vein. As shown in Figure 1, the liver is therefore exposed to potentially harmful substances derived from the gut, including translocated bacteria, ethanol, and endotoxins [1]. One of the key roles of the liver is to rapidly clear these substances from the circulation. TJ proteins, such as zonula occludens, normally seal the junctions between intestinal endothelial cells at their apical aspect and thus have a vital role in preventing translocation of harmful substances from the gut into the portal system [39].

Current literature increasingly supports a role for GM and its mucosal gut interaction in the development of liver steatosis, inflammation and fibrosis [40]. Indeed, it has been shown that TJ disruption in mice and NAFLD patients can increase intestinal permeability and bacterial translocation to the liver through the bloodstream [41,42].

Summing up, GM dysbiosis can damage the intestinal epithelium, increase intestinal permeability and expose the liver to harmful bacterial products [43]. Furthermore, the intestinal mucosa immune system, consisting of a complex network of innate and adaptive cell populations, may itself contribute to maintain a delicate balance with intestinal microbiota, since it establishes the tolerance mechanism on intestinal surface [44].

Recent murine model studies have been carried out to ascertain the role of the intestinal barrier in the pathogenesis and progression of NAFLD [45]. Some of these studies combined high-fat diet (HFD) plus dextran sulfate sodium (DSS)-induced colitis to impair gut barrier integrity during the generation of hepatic steatosis [46]. The degree of macrovesicular steatosis, along with lobular inflammation and hepatic focal necrosis, resulted more severe in the model in which the integrity of the intestinal barrier had been chemically altered vs. those exposed only to a HFD. Portal endotoxin levels were elevated in both models, but they were significantly higher in the DSS+HFD model, suggesting the pivotal role of bacterial translocation in NAFLD progression [47].

In human NAFLD, gut permeability and prevalence of small intestinal bacterial overgrowth correlated with the severity of steatosis but not with steatohepatitis [42]. In another human study, plasma IgG levels against endotoxin were found to be increased in biopsy proven human NASH and progressively increased with NASH grade [48]. These findings suggest a relationship between chronic endotoxin exposure and human NASH severity in which increased permeability drives endotoxemia, which in turn triggers inflammatory cytokine responses and insulin resistance [49].

Among intestinal bacteria, *Escherichia coli*, and *Enterococcus* are more efficient in extraintestinal translocation ability and especially in patients with cirrhosis are an important cause of infections. In a recent observational study, *E. coli* emerged as the predominant bacterium in patients with SIBO and NAFLD [50].

However, one should admit that human studies are limited in that peripheral lipopolysaccharide (LPS) levels might not reflect portal LPS levels and might also change longitudinally over time. In other words, increased gut permeability might expose the liver to deleterious levels of LPS without sufficient LPS escaping liver clearance to produce a detectable marked increase in systemic levels [51].

## 6. Endotoxins and Inflammation

Many microbial cell components, or pathogen-associated molecular patterns (PAMPs) including lipopolysaccharide (LPS, endotoxin), flagellin, lipoteichoic acid, and peptidoglycan may affect the physiology and pathology of their host, mediated by TLR or other pattern recognition receptors. TLR signaling is activated upon pathogen and tissue damage recognition that induces a signaling cascade leading to production of inflammatory cytokines [52]. Additionally, pathogen and damage-associated molecules may induce the formation of a cytoplasmic multi-protein complex termed the inflammasome. Inflammasome signaling has been suggested to contribute to ameliorate fatty liver, whereas its dysfunction or deficiency result in aggravated hepatic inflammatory response, liver damage, fibrosis and cell death [53,54].

The possible association between inflammasome activation and NAFLD development and progression may be explained by hepatic influx of saturated fatty acids and LPS that are abundantly found in the model of HFD mice that may induce inflammasome activation [55]. Notably, LPS has effects beyond the liver and gut. For example, chronic low doses of LPS administered subcutaneously impair fasting glucose and insulin, alter hepatic insulin sensitivity, increase visceral and subcutaneous fat, increase adipose tissue macrophage numbers and raise hepatic triglyceride content [49].

Taken together, alterations of host and gut microbiome interactions through defective inflammasome sensing, disrupted inflammatory response, and dysbiosis play a relevant role in hepatic steatosis and its progression to NASH.

## 7. Bacterial Ethanol

Recent studies showed that elevation of endogenously synthesized ethanol contributes to NAFLD development [56]. The role of ethanol in the GLA homeostasis has recently been proposed from the evidence that its chronic consumption was associated with impairment of intestinal barrier function, and increased permeability for bacterial endotoxins and induction of TLR-dependent signaling cascades in the liver [57]. Alcohol is constantly produced by intestinal microbiota even in the absence of an oral alcohol ingestion [58]. It has been shown that a diet rich in sugar may lead to increased blood alcohol levels, and that endogenously synthetized ethanol is eliminated by the alcohol-dehydrogenase (ADH) pathway in the liver. Moreover, it has recently been shown that pediatric and adult alcohol ingestion-free patients with NAFLD have higher blood and breath alcohol, and also acetaldehyde levels [56]. It seems that altered GM composition plays an important role in increasing fasting blood alcohol levels, even if the precise mechanisms in NAFLD development have not yet been fully understood. Hepatic ADH activity is strongly influenced by IR, a condition typical of NAFLD patients [56,59]. 

Zhu’s group examined GM composition and ethanol levels in the blood of NASH, obese, and healthy children [60]. Only a few differences were evident in the GM composition of NASH as compared with obese patients without liver disease, and included differences across phyla, families, and genera in Proteobacteria, Enterobacteriaceae, and *E. coli*, respectively. Some of these microbiome changes included more alcohol-producing bacteria, associated to a significant increase in ethanol levels in NAFLD subjects as compared to both obese and healthy children. Furthermore, increased levels of ethanol were specifically detected in correlation with NASH. All in all, these results suggest that production of ethanol by GM may serve as a hepatotoxin, contributing to development of NAFLD and its progression to NASH [60].

Summing up, increased permeability, endogenous ethanol and systemic endotoxin concentrations reflect some GLA dysfunction in obesity and its hepatic complications. In this regard, our group recently showed that the lactulose/mannitol ratio values parallels the grade of liver involvement, significantly correlated with ethanolemia and endotoxemia concentrations. Increased permeability was a risk factor for the development of steatosis [61].

## 8. Bile Acids and Farnesoid X Receptor

A possible role of bile acids (BA) in glucose, lipid and energy homeostasis and inflammation through activation of the FXR and G-protein coupled receptor (TGR5) has been recently proposed. Changes in BA pool size and signaling pathways may be convenient in some metabolic diseases [62].

FXR is strongly expressed in the liver and intestine, where it is a regulator of BA enterohepatic circulation. It is known to have a crucial role in hepatic de-novo lipogenesis, VLDL export and plasma trigliceryde turnover [63]. A recent human study showed low FXR protein expression in patients with NASH vs. simple NAFLD, suggesting a protective role of FXR in liver disease progression [64]. However, the FXR seems to have a tissue-specific action: (1) the intestinal FXR antagonism inhibits sterol regulatory element-binding protein-1 (SREBP-1) with positive effects on lipid metabolism; (2) conversely, its hepatic agonism increases insulin-sensitivity, reduces obesity and suppresses inflammation [11,65]. TGR5 instead binds secondary BA and stimulate GLP-1 and peptide tyrosine (TYY) secretion, playing an important role in glucose homeostasis and food intake. BAs influence the composition of GM and in turn they are chemically modified by bacterial enzymes [66]. BAs have anti-microbial properties due to their detergent actions by damaging microbial membranes and intracellular structures. Furthermore, taurine catabolic end-products of BA catabolism promote proliferation of some bacteria species, with a consequent influence of microbial gut composition [62]. 

The enzyme bile salt hydrolase (BSH) of *Lactobacillus* and *Bifidobacteria* spp. catalyzes the deconjugation of bile salts to generate the unconjugated cholic acid (CA) and chenodeoxycholic acids (CDCA), which then are further modified by colonic bacterial 7α-dehydroxylasis to secondary BA, such as deoxycholic acid (DCA) and lithocolic acid (LCA). The 7α-dehydroxylation is catalyzed by microbial enzymes of bile acid inducible (BAI) operon, a system biochemically and genetically characterized in many *Clostridium* species. Therefore, changes in bacterial gut composition which alter the BSH and BAI enzyme copies expression influence bile acid pool and its detergent and signaling properties [67].

These free BA (CA, CDCA, DCA) are less efficient in the solubilization and absorption of lipids from diet and are more largely excreted in feces than conjugated counterparts, with a consequent positive effect on total and low-density lipoprotein (LDL)-cholesterol plasma concentrations [68]. Despite this favorable effect on lipid profile, the unconjugated BA promotes the intestinal FXR signaling. This results in an increased production of ceramides triggering NAFLD through induction of fatty acid synthesis due to SREBP-1 signaling, oxidative stress and mitochondrial damage. Ceramides also impair adipose tissue function through the reduction of beige in favor of white adipocytes [69]. Studies show that the GM modulation by treatment of HFD-fed mice with antibiotics results in decreased adverse metabolic phenotypes probably due to a decrease of *Lactobacillus* spp. and of BSH activity as well. The decrease of the latter resulted in increased levels of tauro-β-muricholic acid (T-β-MCA), a substrate of BSH and a potent FXR antagonist. As has emerged in the literature, mice that were lacking in expression of FXR in the intestine were resistant to HFD-induced obesity, IR and NAFLD [66]. Conversely, hepatic FXR activation seems to have an anti-steatotic effect with an indirect mechanism due to improvement of and lipoprotein transport [70]. Hepatic FXR stimulation attenuates steatosis in rodents and humans, furthermore HFD-fed FXR-null mice more frequently develop fatty liver [67,68]. Role of intestinal FXR in hepato-steatosis however is still controversial, because both agonism and antagonism seems to play a protective role [71] and are also gender-specific [72]. The activation by the FXR agonist fexaramine in mice reduces weight gain and steatosis via fibroblast growth factor 15 (FGF15) signaling [73], conversely also the intestinal FXR antagonism attenuates hepatic steatosis reducing ceramides and SREBP1 signaling [69].

Moreover, in dysbiotic NASH patients the increase of fecal primary BA, primary: secondary BA fecal ratio, and plasma and hepatic BA concentrations may lead to cytotoxicity and explain NAFLD progression [62]. Indeed, in advanced liver disease and cirrhosis, a decrease of total BA inflow from the liver to the gut may cause a shift toward Firmicutes, particularly *Clostridium*, at the expense of other beneficial Firmicutes taxa (i.e., *Lachnospiraceae*, *Roseburia*, *Rumminococcaceae* and *Blautia*). Members of underrepresented taxa are part of normal GM, which are producers of beneficial SCFAs and are involved in conversion of primary to secondary BA. For this reasons in cirrhosis decrement of the total BA pool may unfavorably impact intestinal and systemic inflammation and worsen dysbiosis [74].

Human studies although are still scarce confirm the reciprocal influence of BA and GM and their role in NAFLD pathogenesis, assuming the role of FXR and G-protein receptor [75].

## 9. Fecal Biomarkers

In the last few years a large attention was dedicated to find non-invasive biomarkers of NAFLD/NASH to overcome the need for the diagnostic gold standard tool, i.e., liver biopsy. Colonic bacteria are a source of many metabolic products measurable in fecal samples by gas chromatography-mass spectrometry [76]: this approach appears therefore to provide a useful marker of GM composition and consequently may be used as a surrogate of NAFLD/NASH presence. It seems that more severe liver damage is associated: (1) with changes in the composition of GM and its metabolome; (2) with the intestinal and systemic inflammatory response; and (3) with the metabolic profile of bile acids. GM modifications are reflected in those of its metabolome, and could therefore be an excellent therapeutic target. It will be necessary to deepen the knowledge on the composition of GM and metabolome in patients with NASH in order to more precisely define altered patterns that could be useful for the diagnosis [77]. Notably, a significant increase in fecal ester compounds was already observed in NAFLD adult patients [78] and a unique fecal metabolomics profile with increased level of 2-butanone and 4-methyl-2-pentanonewas found in pediatric NASH [79]. Other extensive studies are needed to define, validate and standardize the fecal metabolomics and its usefulness in non-invasive diagnosis and staging of NAFLD/NASH. We recently showed that urinary metabolome analysis also contributes to defining a metabolic fingerprint of pediatric obesity and related NAFLD, by identifying metabolic pathways/metabolites reflecting typical obesity dietary habits and GM/GLA perturbations [80].

## 10. Therapeutic Prospectives

Hepatopathy of obese children is frequently recalcitrant to the first line-therapy consisting of lifestyle modifications. Therefore, the individuation of new NAFLD treatments is of extreme relevance in clinical practice especially for those patients who are unable to comply with recommended dietary and physical activity changes.

In general, NAFLD therapies target four main pathways: (1) fat accumulation and metabolic stress; (2) oxidative stress and inflammation; (3) fibrosis progression; and finally (4) gut microbiome and GLA. Pending truly effective therapies, in recent years more and more attention has been dedicated to GLA manipulation with studies conducted in animal models and in humans as well. Below we summarize those approaches which may be prove more promising in the next future. Schematically, possible actions on GLA may include GM manipulation through prebiotics (substances useful to the growth of good GM), probiotics (live microorganisms), and synbiotics (combining probiotics and prebiotics in a form of synergism), that appear to reduce intestinal inflammation and improve the epithelial barrier function [7,81,82].

### 10.1. Probiotics

A meta-analytic study showed in NAFLD the efficacy of probiotic therapies in terms of aminotransferases, cholesterol and tumor necrosis factor α (TNF-α) reduction and insulin resistance improvement [83]. Probiotics could improve gut microbiota composition, and downregulate serum LPS and liver *TLR4,* delaying the progression of liver disease [84]. As shown in Table 1, the strain more frequently used belong to genera *Bifidobacteria* and *Lactobacillus* [85,86]. Notably, *Lactobacillus rhamnosus GG* [87] and *VSL#3* [88] showed promising results in pediatric patients with a decrease in serum alanine amino transferase (ALT) in NAFLD vs. controls, significant weight reduction, and amelioration of liver fibrosis [88]. Similar results were recently obtained in a pediatric multi-strain study using another compound of *Lactobacillus acidophilus*, *Bifidobacterium lactis*, *Bifidobacterium bifidum* and *Lactobacillus rhamnosus* [89]. Therefore, probiotics warrant consideration as a therapeutic tool to treat obese children with liver disease in addition to standard lifestyle intervention strategies.

### 10.2. Antibiotics

In animal models, antibiotics treatments attenuate HFD induced gut and liver inflammation [103], probably due to: (1) a decreased intestinal permeability and LPS-mediated *TLR-4* signaling; and (2) an increased abundance of beneficial bacteria [104]. A number of studies carried out with antibiotic therapy in NAFLD/NASH patients have shown divergent results. The macrolide solithromycin reduces hepatocyte ballooning and inflammation in animal models, without any effect on liver fat content, through an LPS-independent mechanism [105]. Polymyxin B and neomycin reduce hepatic steatosis and endotoxin levels in animal models [106,107]. Studies on rifaximin, active on Gram-negative bacteria, show conflicting results in NASH patients ranging from a significant reduction of ALT, endotoxin and IL-10 levels [108] to the inefficacy on fat liver content and ALT levels [109]. Further studies on antibiotics as GLA-modulators are needed, especially to evaluate risks/benefits in light of recent data showing a possible increased risk of obesity due to antibiotic exposure in early life [104,110].

### 10.3. Fecal Microbiota Transplantation

In addition to probiotic supplementation, fecal microbiota transplantation has been shown to attenuate HFD-induced steatohepatitis, through the modulation of GM. Actually, the fecal microbiota transplantation from lean donors to NASH patients is under study in humans (NCT02469272). Despite its routine utilization it is difficult to consider in present clinical practice, the study results will be useful also to better define NAFLD pathogenetic mechanisms [7].

### 10.4. Farnesoid X Receptor Modulators

Last but probably not least, another group of agents is represented by those acting by FXR modulation. Recent promising research has shown the efficacy of the obeticholic acid (OCA), a semisynthetic derivate of chenodeoxycholic acid. This agent, by modulating FXR signaling, improves liver inflammation and fibrosis in NASH adult patients [111] and reduces intestinal inflammation in colitis rodent models [112]. However, patients receiving OCA show unfavorable lipid profile with increased total cholesterol and LDL and decreased high density lipoproteins (HDL), and a higher HOMA (homeostatic model assessment)-IR [112]. *GS-9674*, a synthetic FXR agonist with a more predictable pharmacokinetics in contrast to OCA, is now under study in human NASH [113]. Natural extracts of *Astragali radix*, cycloastragenol and calycosinvia FXR activation attenuates triglyceride accumulation and hepatic fibrosis in NAFLD animal models [114,115]. Other synthetic FXR agonists, *GW4064, INT-767* and fexaramine, showed similar results in obese rodents [66,73,83]. Therefore, modulation of FXR signaling appears to be an emerging therapeutic molecular target for preventing NASH progression [12]. Given still conflicting reports on tissue-specific activity, gender-specificity and negative effect on lipid and glucose profiles, further extensive human studies are needed to better define its efficacy, safety and indications and to design possible selective BA receptor modulators (SBARMs) with minimal side-effects especially in pediatric age.

### 10.5. Anti-LPS Immunoglobulins

A recent promising therapy is oral supplementation of *IMM-124e*, an extract of bovine colostrum rich in IgG obtained from cows immunized versus LPS, that improved liver fat, insulin sensitivity [116] and immune-mediated colitis [117] in animal models, and improved glycemic control in a small pilot human study [118]. Benefits seem to be due to reduction of liver exposition to GM LPS and consequent Kupffer cells activation.

### 10.6. Vitamin D

Finally, a key role in obesity, MetS and NAFLD seems to be played also by vitamin D deficiency, although the underlying mechanism is poorly understood. Recently, the possible involvement of Vitamin D on GLA dysregulation is slowly emerging. In fact, optimal vitamin D levels are essential to maintaining integrity of intestinal permeability, through the up-regulation of TJ components and mucous proteoglycans in the ileum epithelium, and to maintain the gut microbiota in a state of eubiosis, through the expression of specific α-defensins and their converting enzyme (matrix metalloproteinase 7—MMP7) by Paneth cells of intestinal mucosa. The presence of vitamin D deficiency in murine HFD models promotes leaky gut, dysbiosis, endotoxemia, systemic inflammation and consequent IR and liver steatosis [119]. Thus, supplementation with vitamin D has been recommended [120].

## 11. Conclusions

Obesity and obesity-related liver disease (NAFLD/NASH) are major health concerns. At present there is no efficient treatment available for children or adults. Certainly, healthy diet and adequate physical activity levels remain the mainstay treatments in obese patients with hepatic complications, but the individuation of alternative therapeutic targets is critical especially in those with poor compliance to the prescribed lifestyle changes.

Advances obtained in the understanding of the role of GLA in NAFLD pathogenesis, and the encouraging results already obtained by GM modulation via probiotic supplementation appear a presently promising and safe innovative mode of therapy. However, other extensive and long-term studies are needed to better define which are the best probiotic strains, their doses, timing, and duration of supplementation therapy. This will serve to individualize probiotic therapy with a patient-tailored approach for modulating intestinal permeability, endotoxemia, and treating liver disease [15,43,121]. Manipulating bacterial communities by in situ microbiome engineering with high specificity and efficacy (i.e., specifically producing anti-inflammatory or antioxidants molecules) remains still a speculative way, possibly leading to wholly new therapeutic strategies [122].

Finally, FXR modulation through obeticholic acid and similar agents is an encouraging approach needing confirmation in pediatrics.

## Figures and Tables

**Table 1:** Summary of human studies with probiotics and synbiotics in non-alcoholic fatty liver disease.

Reference	Population	Strain	Time	Outcome
Probiotics				
Loguercio et al., 2005 [90]	Adults: 22 NAFLD, 20 alcoholic CIR, 20 HCV, 16 liver CIR (without definition)	*VSL#3* (*Bifidobacteria*, *Lactobacilli*, and *Staphylococcus thermophilus*)	24 wks	Decreased ALT and AST in all groups.
Solga et al., 2008 [91]	4 adult patients with NAFLD	*VSL#3* (*Bifidobacteria*, *Lactobacilli*, and *Staphylococcus thermophilus*)	32 wks	Increased liver steatosis.
Vajro et al., 2011 [87]	20 obese children with NAFLD	*Lactobacillus rhamnosus* GG	8 wks	Decreased ALT and anti-peptidoglycan polysaccharide Abs.
Aller et al., 2011 [92]	28 adult patients with NAFLD	*Lactobacillus bulgaricus* and *Staphylococcus thermophilus*	12 wks	Decreased ALT and GGT.
Mykhal’chyshyn et al., 2013 [93]	72 adult patients with T2D and NAFLD	*Lactobacillus*, *Lactococcus*, *Propionibacterium*, *Bifidobacterium*, *Aceticbacterium*	4 wks	Decreased IL-6, IL-8, TNF-α, IL-1β and IFN-α.
Nabavi et al., 2014 [94]	72 adult patients with NAFLD	*Lactobacillus acidophilus* La5 and *Bifidobacterium breve* subsp. *lactis* Bb12	8 wks	Decreased ALT, AST, TC, and LDL-C.
Alisi et al., 2014 [88]	44 obese children with NAFLD	*VSL#3* (*Bifidobacteria*, *Lactobacilli*, and *Staphylococcus thermophilus* )	16 wks	Improved fatty liver severity, decreased BMI and increased GLP1/aGLP1.
Famouri et al., 2016 [89]	64 obese children with NAFLD	*Lactobacillus acidophilus*, *Bifidobacterium lactis, Bifidobacterium bifidum,* and *Lactobacillus rhamnosus*	12 wks	Decreased ALT; AST, TC, LDL-C, TG. Reduced waist circumference.
Sepideh et al., 2016 [95]	42 adult patients with NAFLD	*Lactobacillus casei*, *Lactobacillus acidophilus*, *Lactobacillus rhamnosus*, *Lactobacillus bulgaricus*, *Bifidobacterium breve*, *Bifidobacterium longum*, and *Staphylococcus thermophilus*	8 wks	Decreased FBS, insulin, IR, TNF-α, and IL-6.
Synbiotics				
Loguercio et al., 2002 [96]	Adults: 12 HCV, 10 alcoholic cirrhosis, 10 NASH	*Lactobacillus acidophilus, Bifidobacterium bifidum, Lactobacillus rhamnosus, Lactobacillus plantarum, Lactobacillus salivarius, Lactobacillus bulgaricus, Lactobacillus casei, Bifidobacterium lactis*, *Bifidobacterium breve,* and FOS.	8 wks	NASH patients: decreased ALT, GGT, and TNF-α.
Malaguarnera et al., 2012 [97]	66 adult patients with NASH	*Bifidobacterium longum* and FOS.	24 wks	Reduced steatosis and NASH activity. Decreased AST, CRP, TNF-α and endotoxin.
Shavackhi et al., 2013 [98]	64 adult patients with NAFLD	*Lactobacillus acidophilus*, *Lactobacillus casei*, *Lactobacillus rhamnosus*, *Lactobacillus bulgaricus*, *Bifidobacterium breve*, *Bifidobacterium longum*, *Staphylococcus thermophilus*, and FOS.	24 wks	Decreased AST, ALT, TGs, TC, BMI and steatosis.
Wong et al., 2013 [99]	20 adult patients with NASH	*Lactobacillus plantarum, Lactobacillus delbrueckii* spp. *bulgaricus*, *Lactobacillus acidophilus*, *Lactobacillus rhamnosus*, *Bifidobacterium bifidum* and inulin.	26 wks	Decreased IHTG content and AST.
Eslamparast et al., 2014 [100]	52 adult patients with NAFLD	*Lactobacillus casei*, *Lactobacillus rhamnosus*, *Staphylococcus thermophilus*, *Bifidobacterium breve*, *Lactobacillus acidophilus*, *Bifidobacterium longum*, *Lactobacillus bulgaricus*, and FOS.	30 wks	Inhibition of NF-κB and reduction of TNF-α.
Ferolla et al., 2016 [101]	50 adult patients with NASH	*Lactobacillus reuteri*, guar gum and inulin.	12 wks	Reduction in steatosis. Decreased weight, BMI and waist circumference.
Mofidi et al., 2017 [102]	50 adult patients with NAFLD and normal/low BMI	*Lactobacillus casei*, *Lactobacillus rhamnosus*, *Staphylococcus thermophilus*, *Bifidobacterium breve*, *Lactobacillus acidophilus*, *Bifidobacterium longum* and *Lactobacillus bulgaricus*, and FOS.	28 wks	Reduction in steatosis and fibrosis. Decreased FBS, TGs and inflammatory mediators.

Abs: antibodies; ALT: alanine aminotransferase; AST: aspartate aminotransferase; BMI: body mass index; CIR: cirrhosis; CRP: C-reactive protein; FBS: fasting blood sugars; FOS: fructo-oligossacharides; GGT: γ-glutamyltranspeptidase; GLP1: glucagon-like peptide 1; IFN: interferon; IHTG: intrahepatic triacylglycerol; IL: interleukin; IR: insulin resistance; LDL-C: low-density lipoprotein cholesterol; NAFLD: non-alcoholic fatty liver disease; NF-κB: nuclear factor kB; T2D: type 2 diabetes; TC: total cholesterol; TGs: triglycerides; TNF-α: tumor necrosis factor α; wks: weeks.
